# New Type of Papillomavirus and Novel Circular Single Stranded DNA Virus Discovered in Urban *Rattus norvegicus* Using Circular DNA Enrichment and Metagenomics

**DOI:** 10.1371/journal.pone.0141952

**Published:** 2015-11-11

**Authors:** Thomas Arn Hansen, Helena Fridholm, Tobias Guldberg Frøslev, Kristín Rós Kjartansdóttir, Eske Willerslev, Lars Peter Nielsen, Anders Johannes Hansen

**Affiliations:** 1 Centre for GeoGenetics, Natural History Museum of Denmark, University of Copenhagen, DK-1350, Copenhagen, Denmark; 2 Department of Autoimmunology and Biomarkers, Statens Serum Institut, DK-2300, Copenhagen S, Denmark; University of Nebraska-Lincoln, UNITED STATES

## Abstract

*Rattus norvegicus* (*R*. *norvegicus*) are ubiquitous and their presence has several effects on the human populations in our urban areas on a global scale. Both historically and presently, this close interaction has facilitated the dissemination of many pathogens to humans, making screening for potentially zoonotic and emerging viruses in rats highly relevant. We have investigated faecal samples from *R*. *norvegicus* collected from urban areas using a protocol based on metagenomic enrichment of circular DNA genomes and subsequent sequencing. We found a new type of papillomavirus, with a L1 region 82% identical to that of the known *R*. *norvegicus* Papillomavirus 2. Additionally, we found 20 different circular replication associated protein (Rep)-encoding single stranded DNA (CRESS-DNA) virus-like genomes, one of which has homology to the replication-associated gene of Beak and feather disease virus. Papillomaviruses are a group of viruses known for their carcinogenic potential, and although they are known to infect several different vertebrates, they are mainly studied and characterised in humans. CRESS-DNA viruses are found in many different environments and tissue types. Both papillomaviruses and CRESS-DNA viruses are known to have pathogenic potential and screening for novel and known viruses in *R*. *norvegicus* could help identify viruses with pathogenic potential.

## Introduction

The Norwegian Rat, *Rattus norvegicus* (*R*. *norvegicus*), commonly known as sewer rat, lives sympatrically with humans in urban and rural areas, and has a long history of being a vector of human pathogens (of e.g. *Leptospirosis* and Hantavirus) [1]. Thus in-depth screening of rats for potentially emerging viruses and known pathogens could prove important as shown by others [2,3], who have found several viruses closely related to human pathogens. Metagenomic studies have become widely used for investigating the microbial composition in human and animal samples with the aim of identifying known and novel viruses and pathogens [2,4,5]. Hence, discovery of intra-family and even more distant related viruses can be done simultaneously. Combining metagenomics with molecular enrichment techniques, like the selective amplification of circular nucleic acids, can be a valuable tool to enhance the detection of specific types of microbes and viruses of interest. Two major groups of viruses with a circular genome arrangement are the papillomaviruses and the Circular replication associated protein (Rep)-encoding single-stranded DNA (CRESS-DNA) viruses.

*Papillomaviridae* constitutes a diverse group of viruses with a circular double-stranded DNA (dsDNA) genome of approximately 8000 bp. Papillomaviruses have two sets of genes expressed at different times during the replication cycle of the virus, i.e. the early genes (E1-7) and the late genes (L1-2). The early genes code for non-structural proteins, whereas the late genes code for structural proteins. Some papillomavirus strains have an extra late gene coding for the L3 protein, which significance, however, remains unknown [6,7]. Papillomaviruses are classified by comparison of sequence data from the cloned L1 gene, where an isolate is considered new if the L1 gene differs more than 10% from the closest known papillomavirus [8,9]. A difference of 2–10 qualifies for a new subtype, and less than 2% a new variant. Based on these criteria, it was suggested that classification should be possible from metagenomic sequencing [8].

Since the first isolation of papillomavirus from skin warts [10], roughly 170 human associated papillomavirus types have been discovered, some of which are oncogenic [8]. In comparison, only 112 papillomaviruses have been discovered in non-human species, but studies indicate that non-human species harbour their own set of species-specific papillomaviruses [11]. Although rodents are the most diverse and one of the biggest groups of mammals [12], only nine rodent-specific papillomaviruses have been discovered and two of those in *R*. *norvegicus* [13,14]. The most recently discovered *R*. *norvegicus* papillomavirus was isolated from laboratory rats and classified in the genus Iotapapillomavirus, and was named *R*. *norvegicus* papillomavirus 2 (RnPV2) [14]. Other rodent papillomaviruses, e.g. the *Mastomys natalensis* papillomavirus, can induce benign skin tumours in the form of papillomas and keratoacanthomas in the multimammate rat *Mastomys coucha* [15]. Given the role of papillomaviruses in cutaneous tumour development in mammals, including humans, characterising novel rodent papillomavirus could provide a new animal model for human cancer research. Simultaneously, by adding to the ever-growing list of viral genomes in public databases, homology searches will be improved, aiding in the detection and identification of novel viruses.

With the advent of metagenomic technologies, the discovery rate of CRESS-DNA viruses has significantly increased [16] and they have been discovered in various environmental, vertebrate and insect samples. The CRESS-DNA viruses–including e.g. the families *Circoviridae*, *Geminiviridae*, *Nanoviridae* and *Microviridae*–,are characterised by having a circular single-stranded DNA genome coding for a replication initiator protein involved with rolling circle replication [17] and small genomes ranging from ~1.7 to ~3 kb [18]. The high success rate of identifying novel CRESS-DNA viruses has outpaced the characterization of their traits and role in pathogenicity. Due to their fast evolving properties, CRESS-DNA viruses constitute a highly diverse group [16]. They are often identified by similarity of the replication protein to that of already known CRESS-DNA and CRESS-DNA-like viral genomes, whereas the less conserved nature of the structural proteins impair similarity based discovery. Another common trait often found in CRESS-DNA virus is a nonanucleotide sequence incorporated into a stem-loop structure [16]. The nonanucleotide sequence has a conserved motif that can vary to some extent. The CRESS-DNA viruses are known to cause several diseases in animals, e.g. beak and feather disease caused by Beak and feather disease virus (BFDV) and the porcine circovirus infecting pigs and causing porcine circovirus associated disease. Rodents have previously been shown to carry several CRESS-DNA viruses and could thus be important vectors, stressing the importance of the characterization of unknown CRESS-DNA viruses to establish any zoonotic incidents and potential.

By employing a method for enrichment of small circular DNA genomes in combination with subsequent high-throughput sequencing, we describe a framework for screening rodents and identifying novel viruses. In this study, we detected the full genomes of 2 viruses, of which one is a new type of papillomavirus named *R*. *norvegicus* papillomavirus 3 (RnVP3), and another is a novel CRESS-DNA virus. Furthermore 19 putative virus contigs resembled known CRESS-DNA viruses, and had a a circular arrangement and a stem-loop containing the canonical nonanucleotide sequence, but with seemingly short genomes. Interestingly, the genomes of the CRESS-DNA virus, RnVP3 and RnPV2, were present in *R*. *norvegicus* at multiple sampled locations across the world.

## Materials and Methods

### Samples

Faecal samples were collected from Malaysia (n = 5) and Denmark (n = 5). The samples collected in Malaysia were shipped at ambient temperature and frozen upon arrival. The samples from Denmark were frozen at –20°C within 24 hours of collection. As the samples are faecal matter left by *R*. *norvegicus*, there has been no handling of animals in this study. Faecal matter was collected in public areas, or by Rentokil, professional pest controllers with permission to remove all rat faecal matter. There has been no sampling of endangered animal or sample collection from protected wildlife habitat and no animals were hurt during this study.

### Enrichment of small circular nucleotide sequences

Each faeces pellet was dissolved in 500 μl of PBS. Two different kits were employed for DNA extraction from the dissolved pellets, the QIAamp DNA Stool for Pathogen detection and the QIAamp DNA Mini and Blood (Qiagen), according to the manufacturers' instructions. The DNA was subsequently passed through a MinElute spin column (Qiagen) and eluted in 20 μl EB buffer. DNA concentration was measured on the Qubit® 2.0 Fluorometer (Invitrogen).

The DNA extracts from each sample where digested with 15 U of Plasmid-Safe™ ATP-dependent DNase (Epicentre) in the presence of 112.5 nmol ATP. The remaining DNA was amplified using the REPLI-g® Midi kit (Qiagen) according to the manufacturers' instructions. A total of 2 μg of DNA was fragmented on the Bioruptor® NGS (Diagenode) to an average length of 300 bp, as verified by running the samples on a High Sensitivity chip on the Agilent 2100 Bioanalyzer instrument. Libraries were built with the NEBnext® DNA Library Prep Master Mix Set for 454 (New England Biolabs) kit, with some modifications. Paired-end sequencing was performed on the Illumina HiSeq 2000 instrument.

### Analyses

Illumina HiSeq FASTQ files were de-multiplexed using the program Novobarcode (http://novocraft.com/main/index.php). The FASTQ files were trimmed based on low quality scores and overlapping paired reads were merged using AdapterRemoval (—trimqualities—trimns—collapse—qualitybase 64) [19]. The reads were subsequently assembled into contigs using Ray Méta [20] (default settings) and contigs were compared to the non-redundant nucleotide and protein database from NCBI using BLASTn and BLASTx respectively, with default settings.

The majority of the metagenomic assemblers lack the option for assembling circular contigs. As observed for assemblies with Illumina reads with the IDBA-UD assembler, contigs of circular DNA seem to be built with identical 3´and 5´ ends [21]. We observed the same sequence pattern at the 3´ and 5´ ends of contigs derived from circular DNA templates assembled with Ray Méta [20], and the 5´ end was manually removed. One contig showed an extensive GTGT low complexity region and this region was truncated prior to removing the overlapping ends. Sequences without unique 3´and 5´ ends were not considered full genomes and discarded, except for papillomavirus sequences. The multiple sequence alignment of papillomavirus genomes RnPV3, RnPV2 and RnPV2 variants was done using Muscle, version 3.8.31 [22], and identities were calculated in a sliding window manner. Circular plots were made using Circos [23], and folding was done using the mfold webserver (http://mfold.rna.albany.edu/?q=mfold/DNA-Folding-Form) DNA folding form [24].

Comparison of the RnPV3 L1 gene with known papillomavirus L1 genes was initially done using the PAVE L1 taxonomy tool and subsequently with MAFFT (online version 7 http://mafft.cbrc.jp/alignment/server/) with the settings E-INS [25] (mafft—thread 3—reorder—maxiterate 1000—retree 1—genafpair input). The resulting alignment consisted of 1944 positions, and contained several ambiguously aligned regions. Gblocks v. 0.91 [26] was employed to produce an alignment of the conserved regions, and resulted in a 1064 nucleotide long alignment. A maximum likelihood phylogeny estimate of the resulting alignment was produced with the rapid bootstrap function in RAxML [27] with 1000 bootstrap replicates (raxmlHPC -f a -m GTRGAMMA -p 12345 -x 12345 -# 1000). The ORFs E1, E2, E6, E7, L1, L2 and L3 were predicted by using ORF finder [28] and E4 was predicted using SIXPACK [29].

Using ORF Finder [28], the ORF for the replication-associated protein was predicted and compared to a custom database of replication-associated proteins of circoviruses and Beak and feather disease viruses using the Sequence Demarcation Tool [30]. Using getorf with the option to check for ORFs crossing circular breakpoint we found the ORFs in the presumable defective or multicomponent viruses.

## Results and Discussion

### Discovery of novel viral sequences

We applied a preferential amplification of circular templates and metagenomic sequencing of *R*. *norvegicus* faecal material in order to screen for novel viruses. The retrieved metagenomic sequences were assembled into contigs and aligned using BLASTn to NCBIs nucleotide database. We uncovered the genome of a new type of papillomavirus. The sequence was deposited to the ICTV, where it was assigned the name *R*. *norvegicus* papillomavirus type 3 (RnPV3), which will be used throughout this text for this sequence. This genome was characterised with predicted ORFs, and the L1 gene was phylogentically compared to all known L1 papillomavirus genes and showed 82% identity to RnPV2 on the nucleotide level. Additionally, we identified the full genome of a variant of RnPV2 in four samples collected from different locations around the world displaying 99% identity to RnPV2. An *in silico* translated alignment of RnPV3 L1 sequence to the RnPV2 L1 protein showed 97% similarity, suggesting that the coding region is conserved at the protein level, despite the changes at nucleotide level. From the translated BLASTx alignments of the generated contigs to NCBIs non-redundant protein database, we additionally found a novel CRESS-DNA virus genome in eight out of ten samples. This CRESS-DNA virus genome was initially recognised by its homology of 30% on amino acid level to the replication gene of BFDV, and further analyses revealed the typical stem-loop containing the eight nucleotides of the canonical conserved nonanucleotide sequence known from circoviruses. Lastly, we discovered 19 other potential candidates of CREES-DNA virus genomes from the BLASTx alignments, all containing the canonical nonamer included in a stem-loop structure.

### Genomic organisation of the new type of papillomavirus

**Fig 1:**
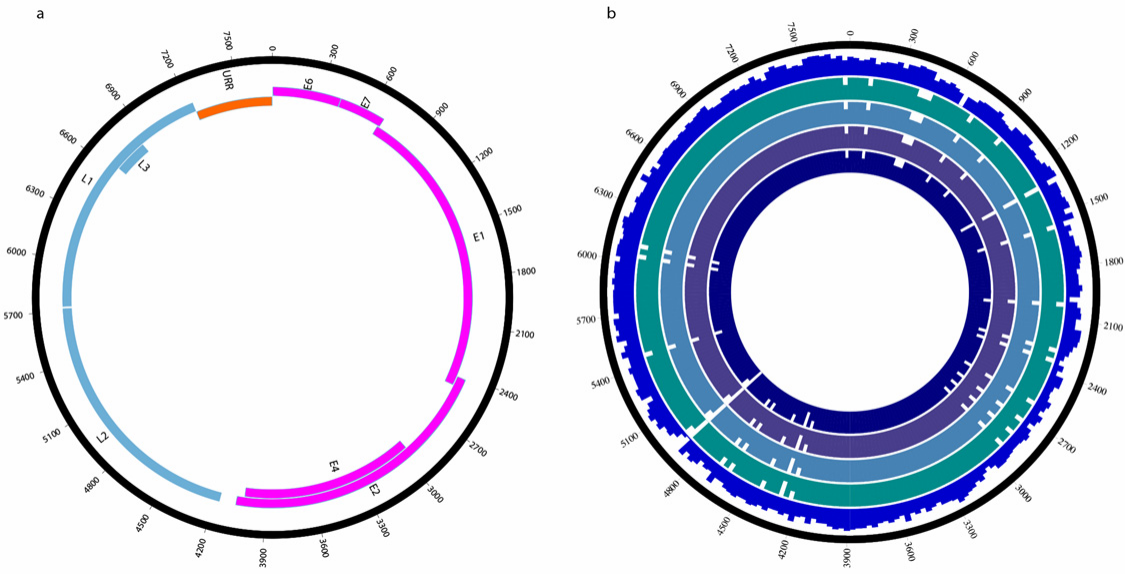
(A) Genome of the putative RnPV3 virus and predicted ORFs. Early genes are shown in magenta, late genes in cyan and the upstream regulatory region (URR) in orange. (B) Multiple alignment of the identified RnPV2 type variants and RnPV3 sequences to RnPV2 (Acc. Nr; HQ625441) genome. From inside out, 4 near-identical RnPV2 type variants, RnPV3 and the outermost track line represents the length of the multiple alignment.

Using our metagenomic approach, we chose to focus on those sequences that could be assembled to a full genome with expected genomic organisation and structure. The assembled genome of RnPV3 is 7707 nucleotides long and has a GC content of 50% equivalent to the GC content of 51% for RnVP2. In the RnPV3 genome, we found seven canonical papillomavirus open reading frames (ORFs), namely E1, E2, E4, E6, E7, L1 and L2 (Fig 1A).

In addition, we found another putative RnPV3 ORF mapping with low homology to the putative L3 protein of Xipapillomavirus 1 [31].

The comparison of the RnPV3 genome and the RnPV2 genome suggested that nearly all regions differed on nucleotide level between the two (Fig 1B). We also found four variants of the RnPV2 genome that showed very high sequence identity (99%) to the known RnPV2 genome. Furthermore, we found contigs that align with high identity to RnPV3 in all our samples collected from different locations of the world (S1 Fig and S1 Text).

**Fig 2:**
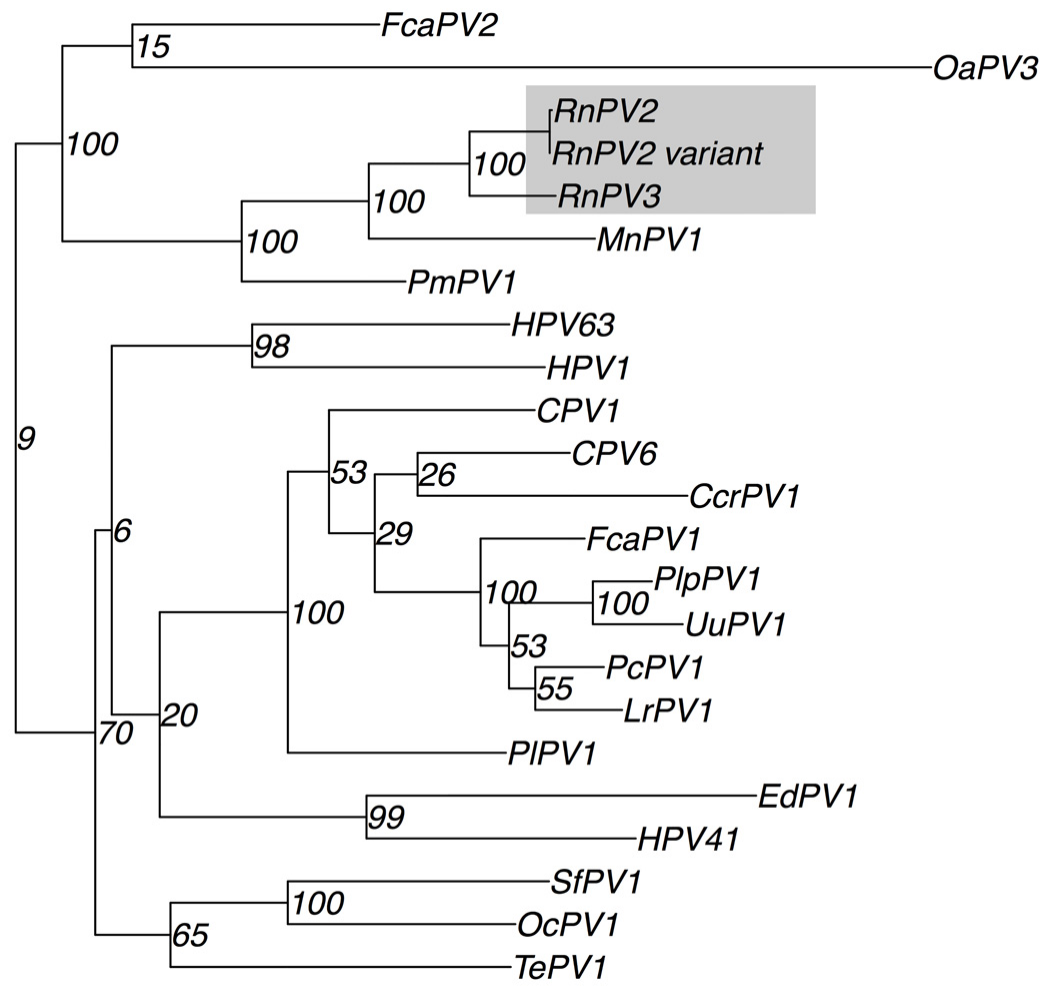
Subset of phylogenetic relationship among the papillomavirus L1 genes and L1 genes from the RnPV3 and RnPV2-variants identified in this study. The bootstraps values presented were inferred from 1000 bootstrap replicates. See S2 Fig for a complete tree of 289 L1 genes, the RnPV3 L1 gene and the RnpV2 type variant L1 genes.

Papillomaviruses are classified phylogenetically based on their L1 sequence [8,9]. Maximum-likelihood based phylogenetic analysis of the putative RnPV3 L1 sequence along with the curated papillomavirus L1 genes of 289 papillomaviruses, including both human and animal papillomaviruses, showed that the RnPV3 L1 sequence clusters closely with the RnPV2 L1 sequence (Fig 2, S2 Fig, and S2 Text) with a bootstrap value of 100.

This indicates that the RnPV3 should be classified as an Iotapapillomavirus like the RnPV2, which was classified by inference of assembled predicted amino acids [14]. RnPV2 has until now been the only member of Iotapapillomaviruses known to infect *R*. *norvegicus*. Due to the fact that RnPV3 clusters with Iotapapillomavirus it would be interesting to study their pathogenicity in *R*. *norvegicus* and to see if such an infection is a transient infection or if it can cause malignancy in the rats. The reference strain for Iotapapillomaviruses is *Mastomys natalensis* papillomavirus (MnPV) (acc. Nr NC_001605) and the group also includes *Peromyscus* papillomavirus 1 (acc. Nr JF755418). It would be very interesting to look at divergence times of the Iotapapillomaviruses and their hosts. Determination of the divergence could help establish the rodent’s evolutionary path and geographical distribution pattern.

We have shown that by using a method targeting circular DNA it is possible to discover a new type of papillomavirus in *R*. *norvegicus*, and thus expanding the diversity of known papillomaviruses in rodents. Conducting an extensive study of the *R*. *norvegicus* papillomavirus virome, by large scale multi-tissue circular DNA enrichment and metagenomic approach, would allow a species-specific set of *R*. *norvegicus* papillomaviruses to be generated adding to the large spectrum of papillomaviruses. Also, it would reveal if the RnPV viruses can become integrated into the host/rat genome and thereby harbour an oncogenic potential as seen by certain of the human papillomaviruses. Thus would also render them interesting as a model virus for the study of virus-induced malignancies. Simultaneously, in depth characterization of the papillomavirus virome of *R*. *norvegicus* could characterize new papillomaviruses which could be used in an animal model system and expose the principal course of infection of papillomaviruses and proof of concept in vaccine development, as for the *Mastomys coucha* and MnPV model system [32,33]. Lastly, ongoing screening for viruses in mammals living sympatric with humans in urban areas will facilitate identification and classification of emerging viruses with potential zoonotic abilities. Screening for different viruses in other mammals living in close association with humans allows us to have a preparedness and knowledge of the surrounding virome; and should a zoonotic event take place, we can detect and identify the source of the infection and take preventive measures.

### CRESS-DNA virus identification

**Fig 3:**
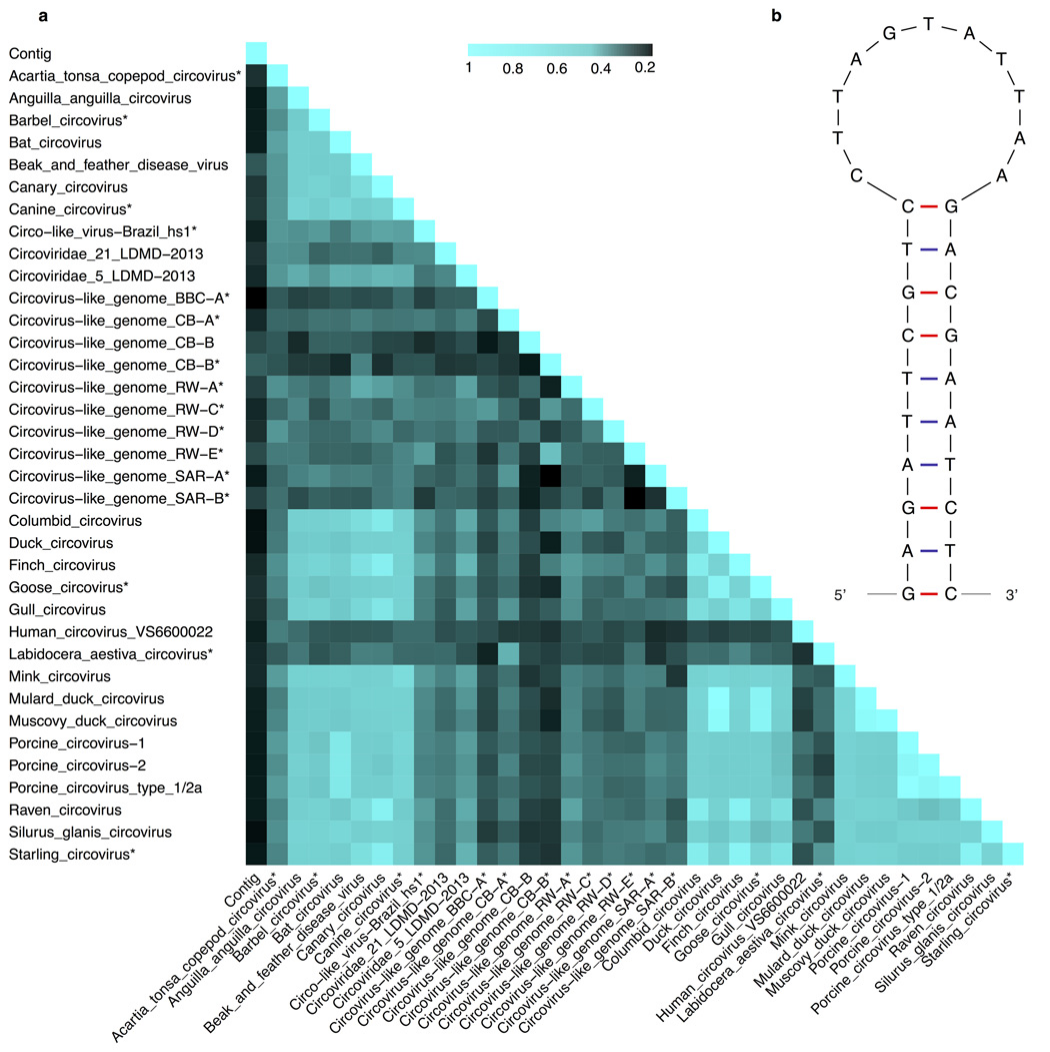
(A) Sequence identities, as calculated by SDT, inferring the homology between circovirus replication-associated proteins, BFDV replication-associated protein and the CRESS-DNA virus genome replication-associated protein discovered in this study. * Putative ORFs. (B) Predicted folding of the TAGTATTAA nonamer and the flanking region from the CRESS-DNA virus discovered in this study, creating the characteristic stem-loop structure.

The metagenomic analyses identified 51 CREES-DNA viral candidates by comparing the contigs to the non-redundant protein database. In 20 of these contigs we discovered a canonical nonamer TAGTATTAC or a variant TAGTATTAA embedded in a stem-like structure. Of these 20 contigs 19 appeared to be circular with overlapping end sequences and approximately 850 nucleotides long. Even though these contigs have a stem-like structure with the canonical nonamer and is presumably circular, they encode 1–4 ORFs larger than 300 nucleotides, and might not be true CRESS-DNA viruses, but are more likely defective molecules or part of multicomponent viruses. The last of the 20 CRESS-DNA virus-like contigs was approximately 1890 nucleotides long contig, found in eight out of ten samples, aligning to BFDV. Translated BLAST alignments of these contigs to the non-redundant protein database resulted in alignments with 30% identity to the BFDV replication-associated protein. Single-stranded circular viral genomes have previously been discovered based on similar low homology to replication-associated genes [34]. It is known that the structural proteins in CRESS-DNA viruses are highly diverse on the amino acid level and difficult to identify using similarity searches like BLAST [16], which probably is the reason why these genes remain undetected in the sequence data. The eight BFDV-like virus genomes detected are completely identical (100% identity) except for a low-complexity region that differed in length located in the replication-associated gene. The translated protein sequence of this new CRESS-DNA sequence has very low identity to any known circovirus, which can complicate and flaw a classic multiple sequence alignment based phylogenetic analysis. Thus we used the species demarcation tool (SDT) [30] to infer the identity by pairwise alignment. The same ORF was predicted in six of the CRESS-DNA viruses and was used in comparison of similarity to 36 replication genes of circovirus including BFDV (Fig 3A).

The novel replication-associated coding sequence had a very low homology to any known CRESS-DNA virus but this analysis showed that the new sequence was similar to circovirus-like genome CB-B (29%) but also clearly similar to BFDV (28%). We detected the signature nonamer sequence TAGTATTAA enclosed in a stem-loop structure with the sequence CGAGATTCGTCCTTAGTATTAAGACGAATCTCG in all eight contigs (Fig 3B). The nonamers from these eight were all identical and differed from the more commonly studied TAGTATTAC motif only in the last nucleotide. In general the nonamer is considered a conserved region, but it is not unusual to find minor differences in the nonanucleotide sequence [16,35]. The discovery of virus genomes is shifting towards using metagenomic approaches, and the CRESS-DNA we present here describes not only the presence of CRESS-DNA virus-like nucleic acids from metagenomic sequencing, but also several of the features known from previous described CRESS-DNA virus. The distant relationship of the novel CRESS-DNA virus to known viruses could be caused by a general lack of discovered mammalian CRESS-DNA viruses. In general, CRESS-DNA viruses are associated with several cases of disease, *i*.*e*. the postweaning multisystemic wasting syndrome, Beak and feather disease and chicken infectious anaemia. Therefore, further investigation of CRESS-DNA viruses should be conducted to shed a light on their connection to diseases, zoonotic potential and their role in the ecosystems.

## Supporting Information

S1 Fig Plot of contigs from all samples mapped to RnPV3. (PDF)

S2 Fig The phylogenetic relationship of the papillomavirus L1 genes. (PDF)

S1 Text Figure text for S1 Fig. (DOCX)

S2 Text Figure text for S2 Fig. (DOCX)
