# Supplementary figures and images for: New Type of Papillomavirus and Novel Circular Single Stranded DNA Virus Discovered in Urban Rattus norvegicus Using Circular DNA Enrichment and Metagenomics
Source: PLoS One. 2015 Nov 11;10(11):e0141952. doi: 10.1371/journal.pone.0141952 (PMC4641689; doi:10.1371/journal.pone.0141952)

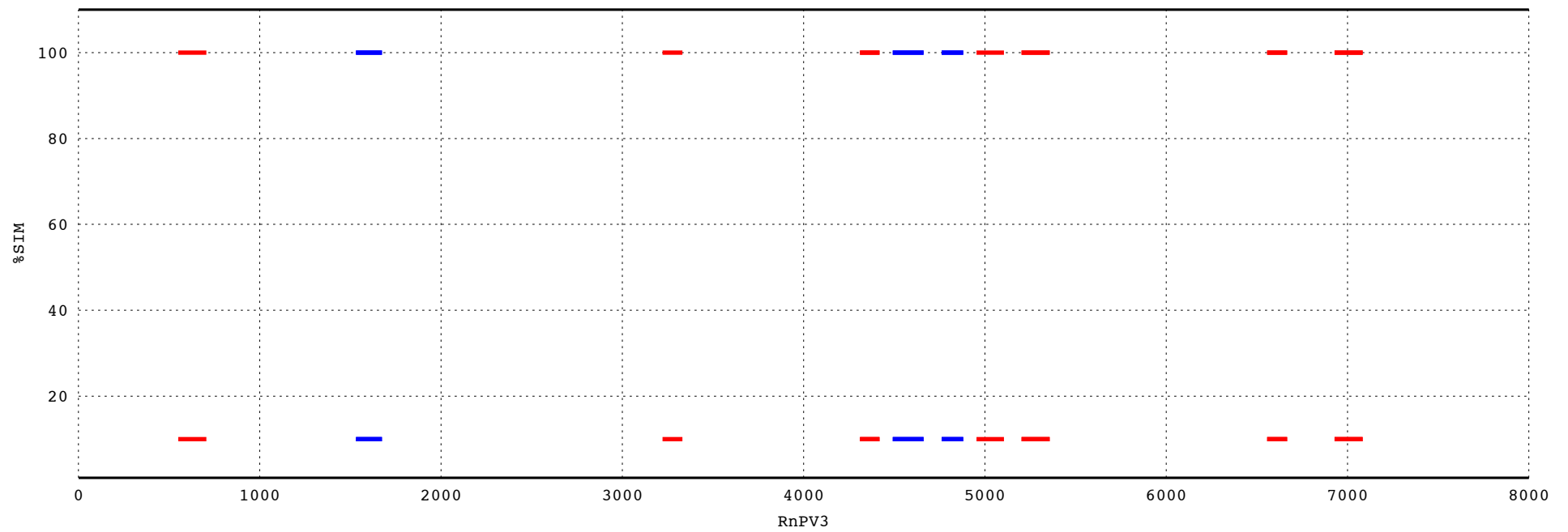

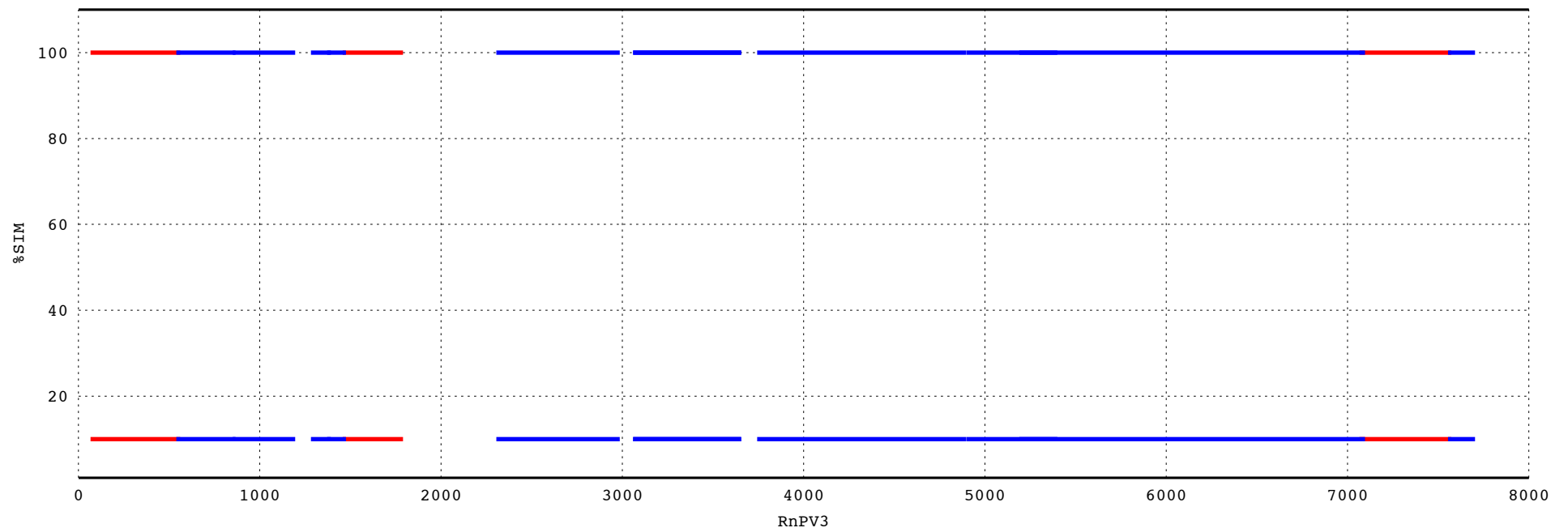

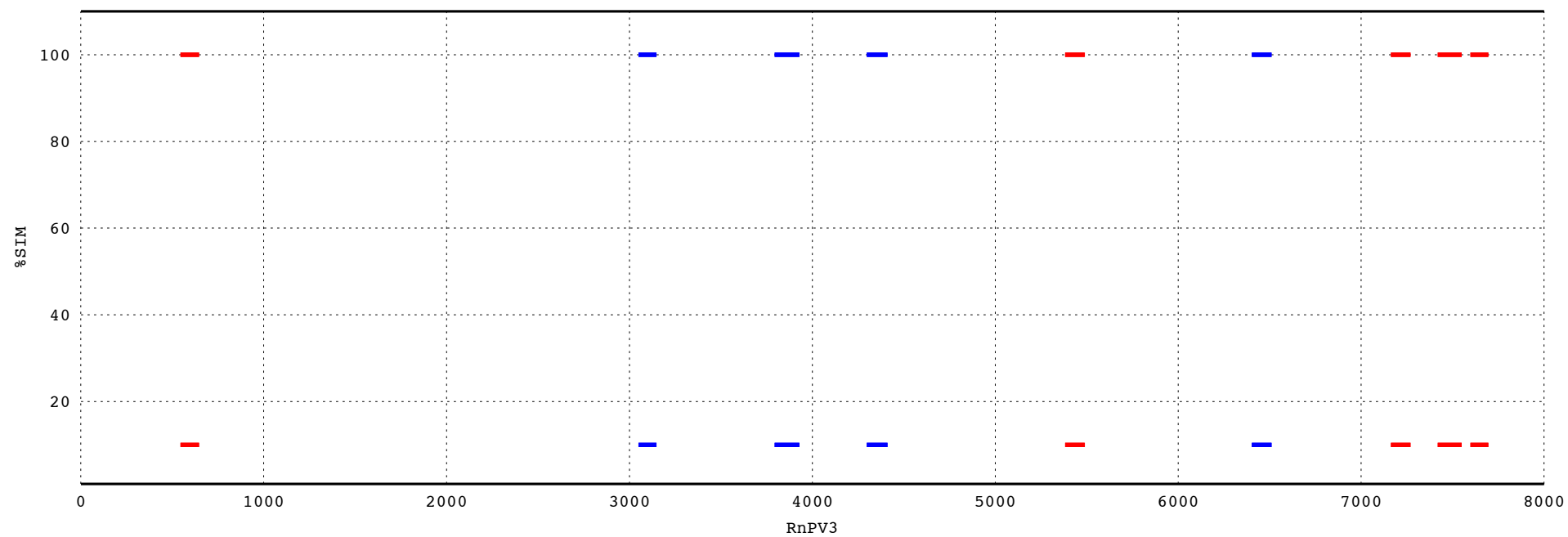

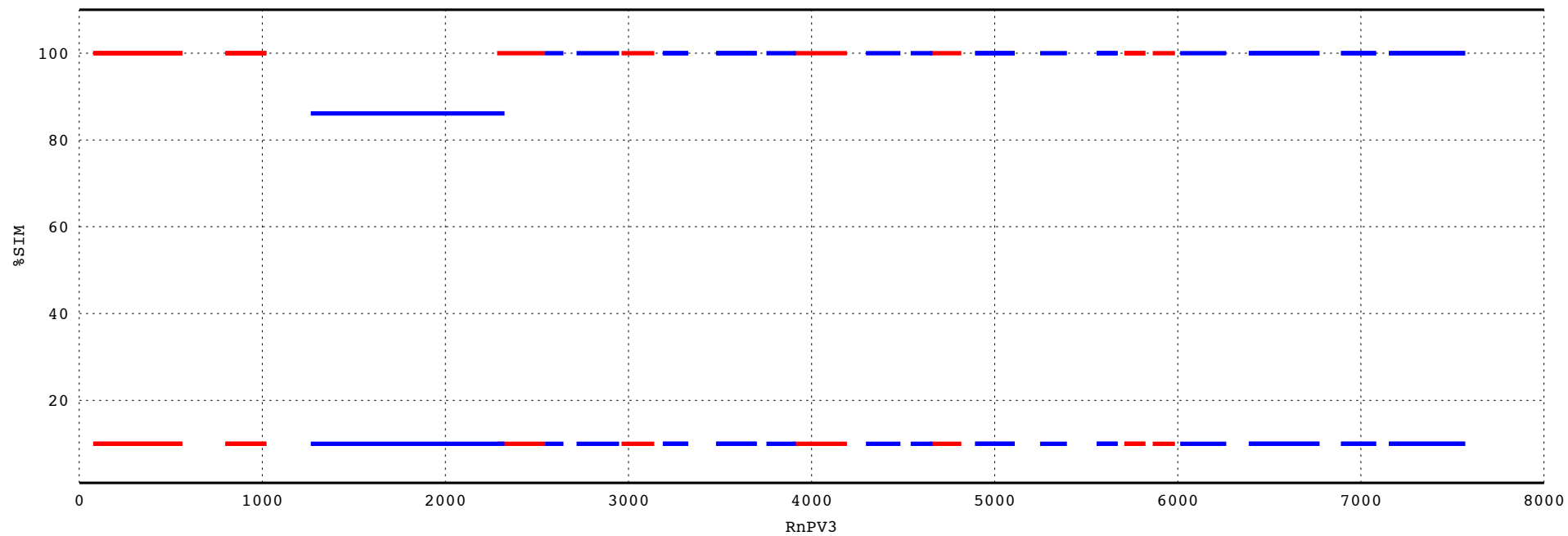

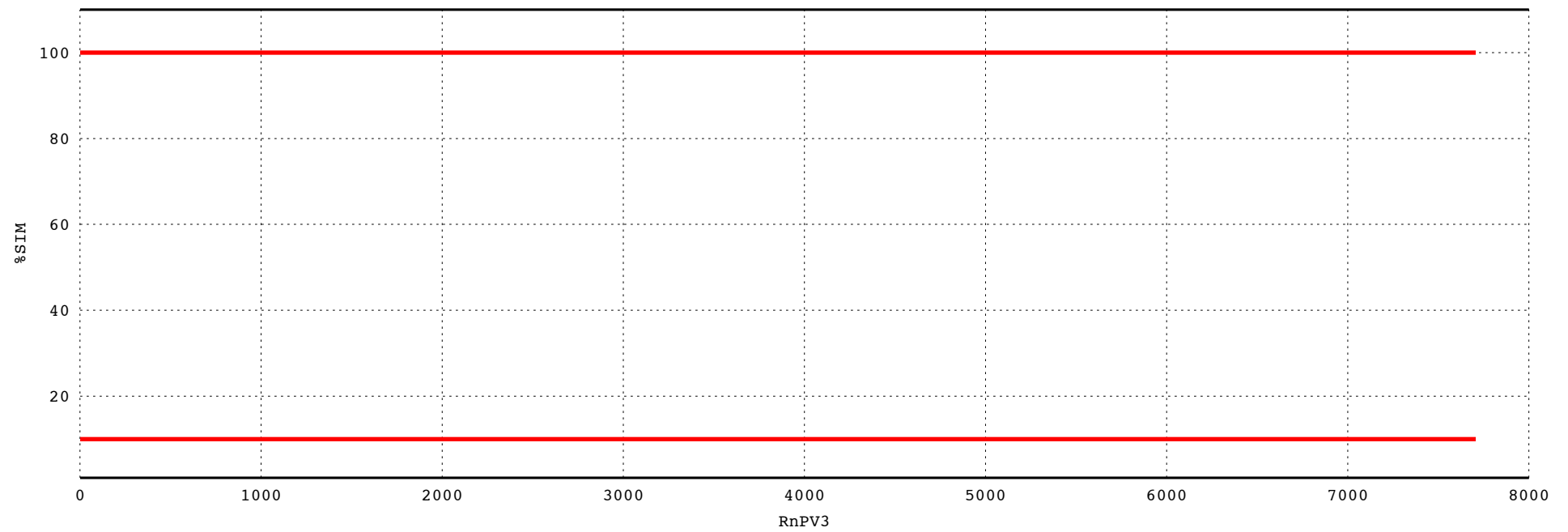

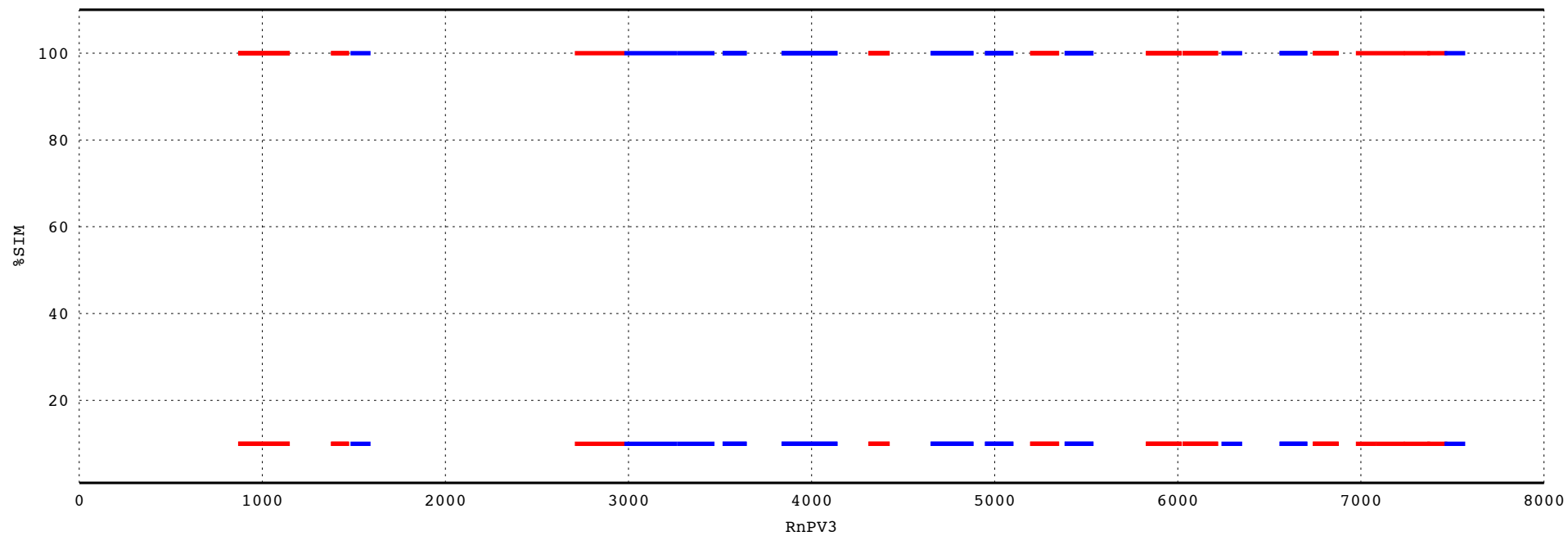

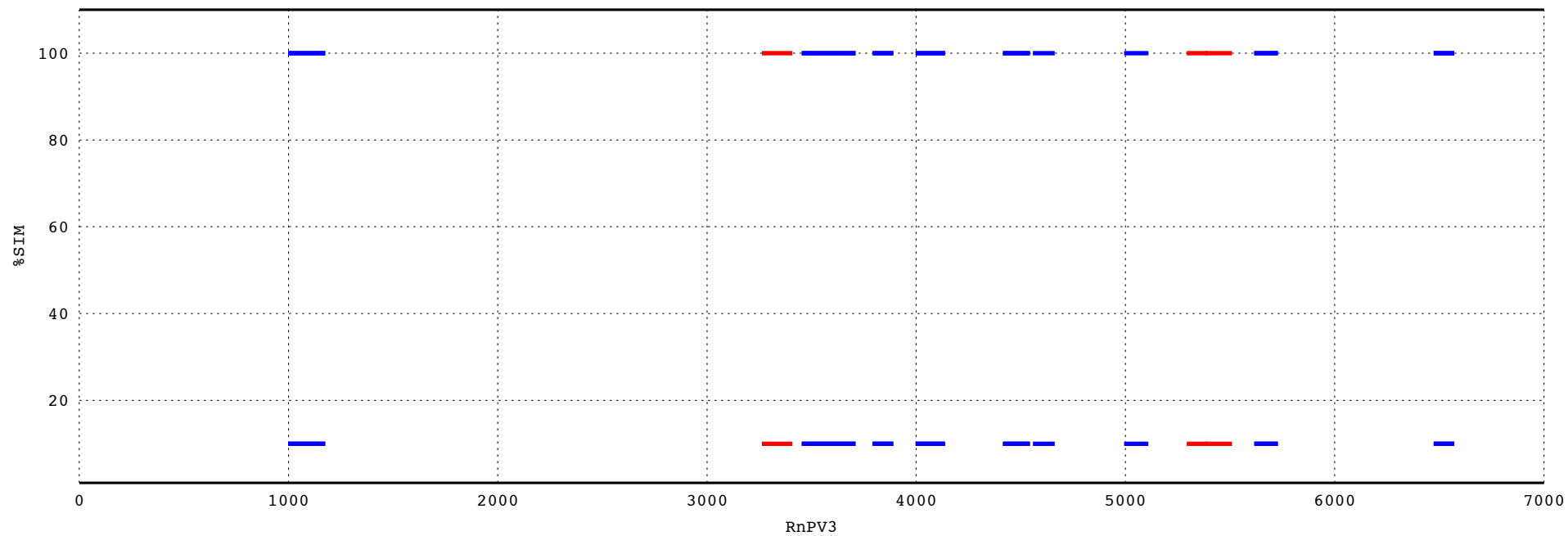

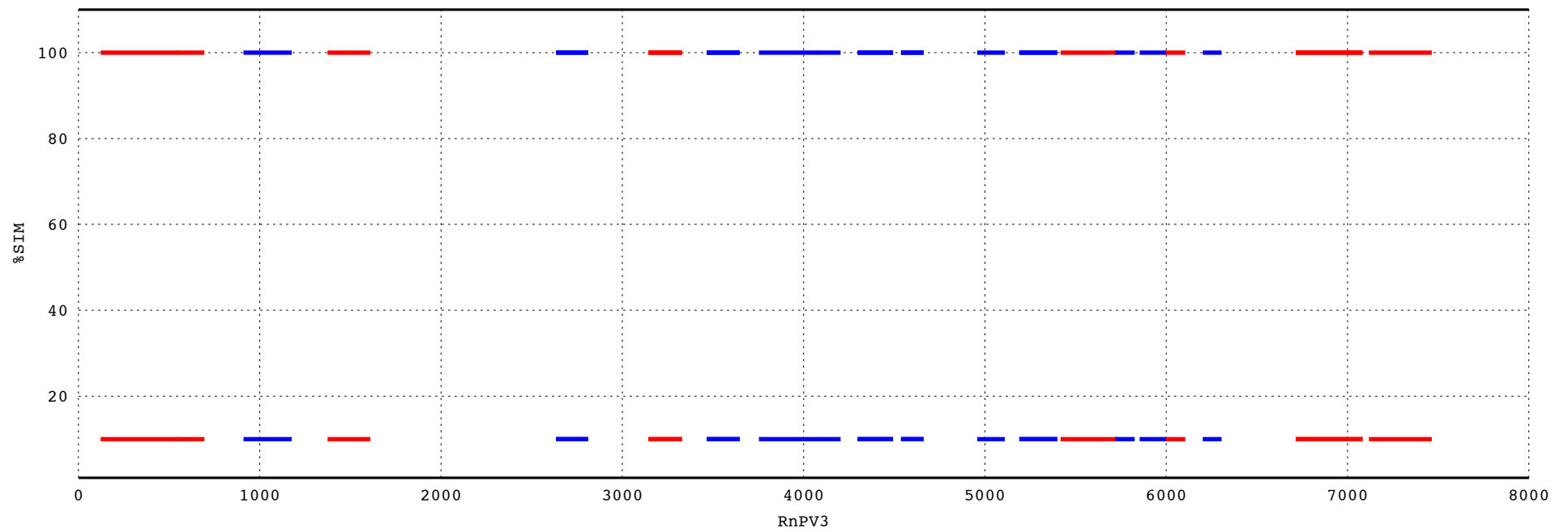

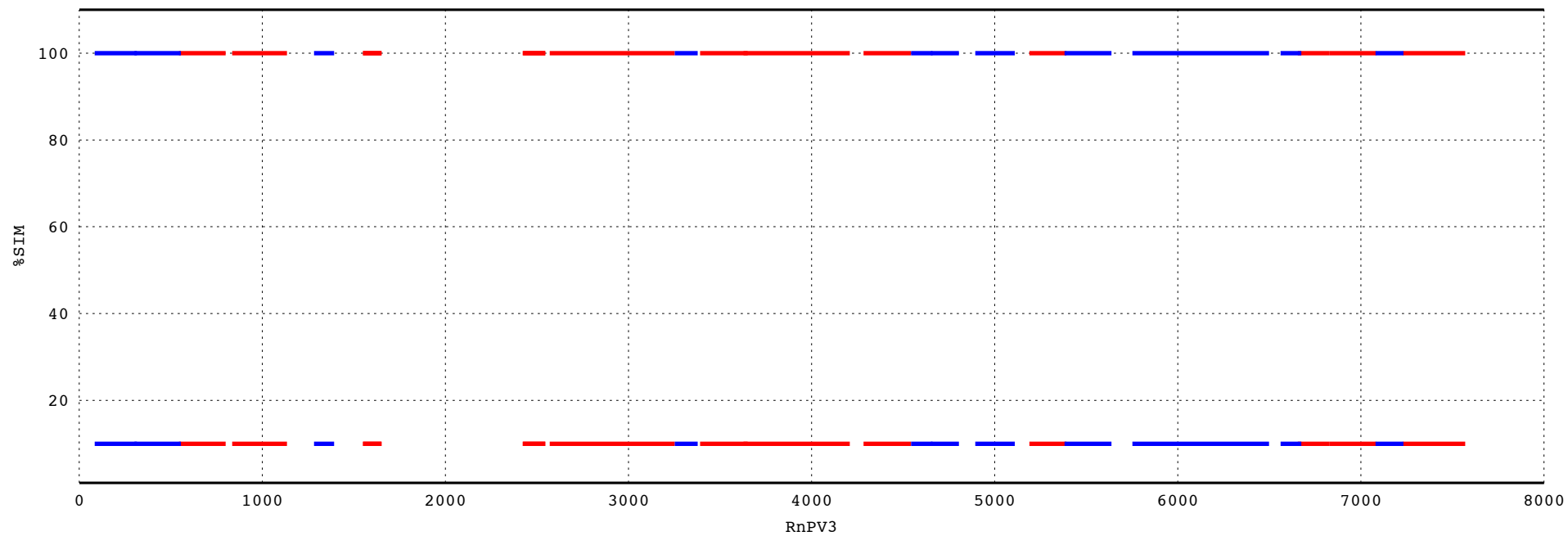

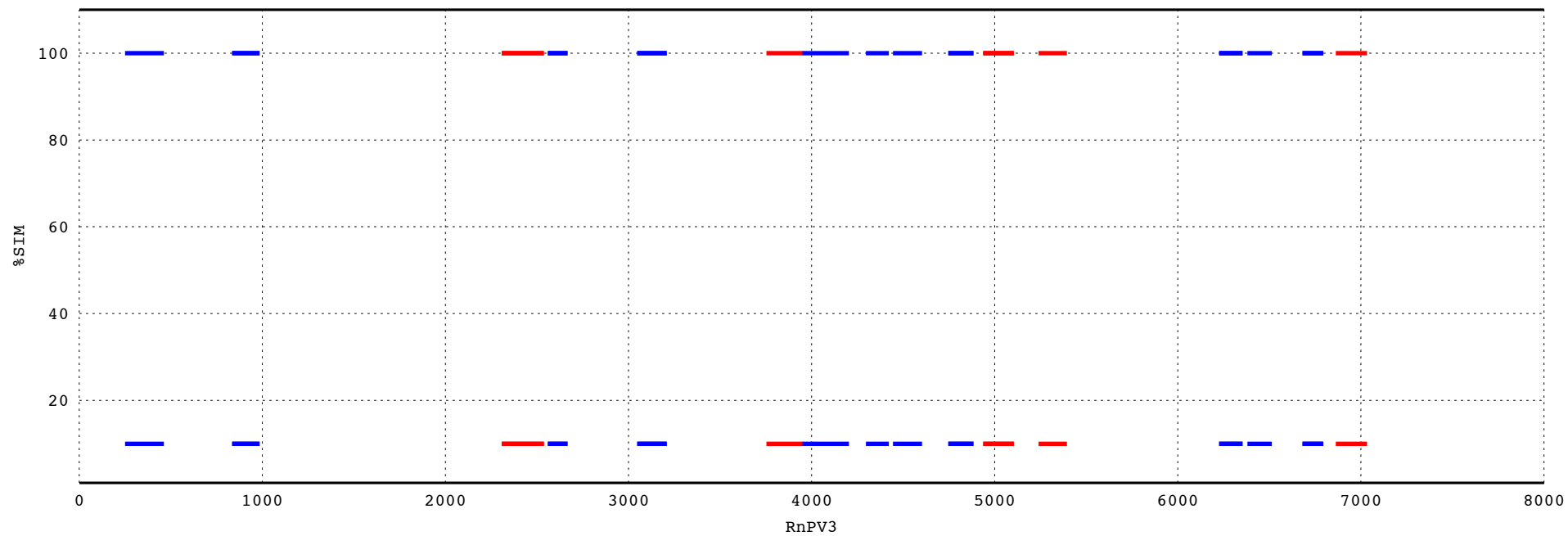

Supplement: S1 Fig — (PDF) [file pone.0141952.s001.pdf]

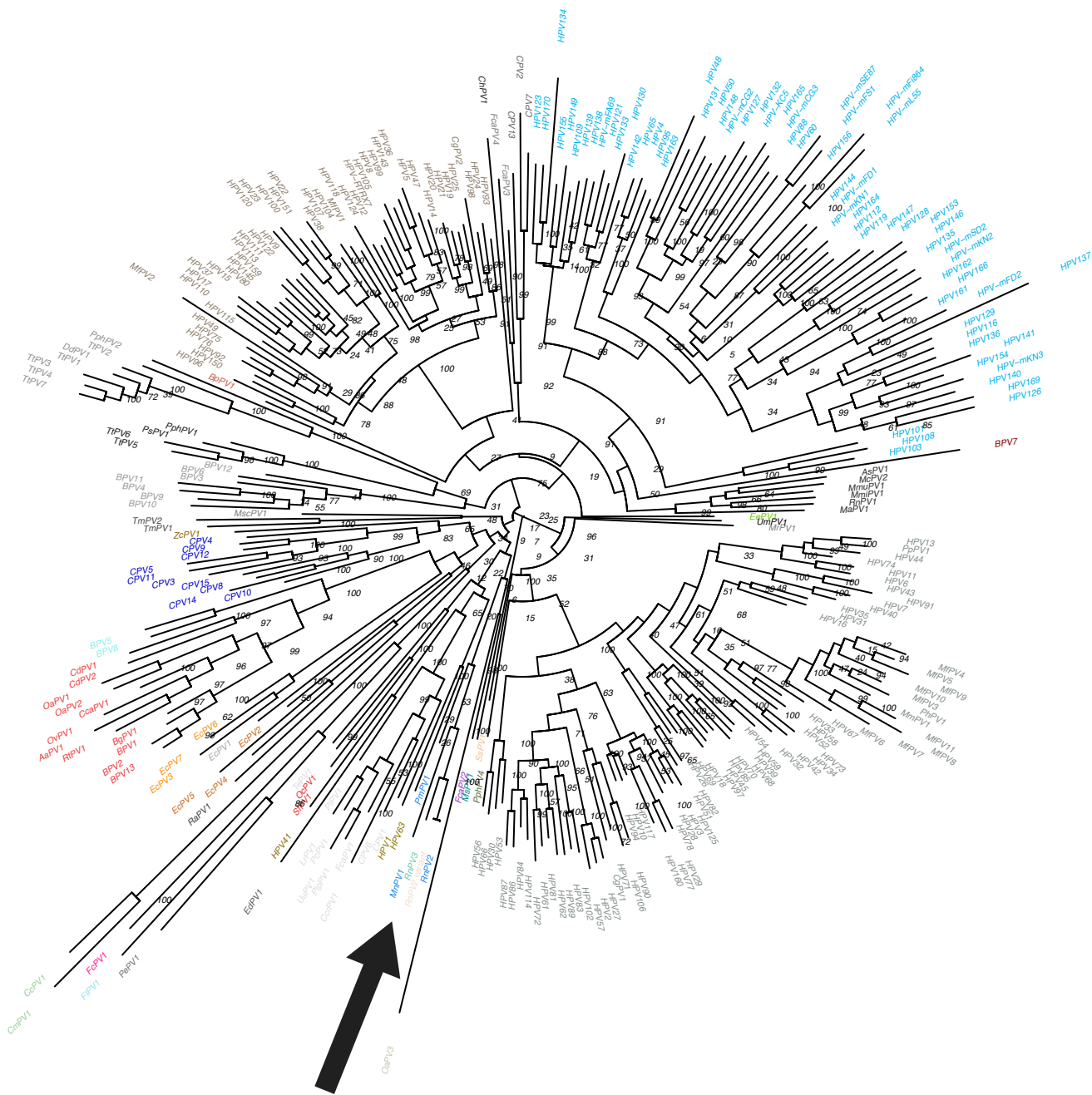

Supplement: S2 Fig — (PDF) [file pone.0141952.s002.pdf]
