# Supplementary material for: New Type of Papillomavirus and Novel Circular Single Stranded DNA Virus Discovered in Urban Rattus norvegicus Using Circular DNA Enrichment and Metagenomics
Source: PLoS One. 2015 Nov 11;10(11):e0141952. doi: 10.1371/journal.pone.0141952 (PMC4641689; doi:10.1371/journal.pone.0141952)
Supplement: S1 Text — (DOCX) [file pone.0141952.s003.docx]

**S1 Text.**

The contigs from different locations were mapped to RnPV3 and the length and per cent identity was plotted. % SIM=Similarity and on the Y-scale the nucleotide position in the RnPV3 genome.
