# Supplementary material for: New Type of Papillomavirus and Novel Circular Single Stranded DNA Virus Discovered in Urban Rattus norvegicus Using Circular DNA Enrichment and Metagenomics
Source: PLoS One. 2015 Nov 11;10(11):e0141952. doi: 10.1371/journal.pone.0141952 (PMC4641689; doi:10.1371/journal.pone.0141952)
Supplement: S2 Text — (DOCX) [file pone.0141952.s004.docx]

**S2 Text.**

The phylogenetic relationship of the L1 genes of 289 papilloma virus from the PAVE database, the RnPV3 and the RnPV2 type variant identified in this study.
